# Circadian and sex differences in carotid-femoral pulse wave velocity in young individuals and elderly with and without type 2 diabetes

**DOI:** 10.3389/fcvm.2022.952621

**Published:** 2022-09-08

**Authors:** Alessandro Gentilin, Paolo Moghetti, Antonio Cevese, Anna Vittoria Mattioli, Federico Schena, Cantor Tarperi

**Affiliations:** ^1^Department of Neuroscience, Biomedicine, and Movement Sciences, University of Verona, Verona, Italy; ^2^Italian Institute for Cardiovascular Research (INRC), Bologna, Italy; ^3^Section of Endocrinology, Diabetes and Metabolism, Department of Medicine, University of Verona and Azienda Ospedaliera Universitaria Integrata Verona, Verona, Italy; ^4^Surgical, Medical and Dental Department of Morphological Sciences Related to Transplant, Oncology and Regenerative Medicine, University of Modena and Reggio Emilia, Modena, Italy; ^5^Department of Clinical and Biological Sciences, University of Turin, Turin, Italy

**Keywords:** aging, diabetes mellitus, arterial stiffness, cardiovascular disease, risk factors, sympathetic activation, sex differences, circadian changes

## Abstract

The incidence of cardiovascular events is higher in the morning than in the evening and differs between sexes. We tested the hypothesis that aortic stiffness, a compelling cardiovascular risk factor, increases in the morning than in the evening in young, healthy individuals between 18 and 30 years (H18–30) or in older individuals between 50 and 80 years, either healthy (H50–80) or with type 2 diabetes (T2DM50–80). Sex differences were also investigated. Carotid-femoral pulse wave velocity (cf-PWV) recorded via Doppler Ultrasound, blood pressure and heart rate were checked at 6 a.m. and 9 p.m., at rest and during acute sympathetic activation triggered by handgrip exercise. Cf-PWV values were lower in the morning compared to the evening in all groups (*p* < 0.01) at rest and lower (*p* = 0.008) in H18–30 but similar (*p* > 0.267) in the older groups during sympathetic activation. At rest, cf-PWV values were lower in young women compared to young men (*p* = 0.001); however, this trend was reversed in the older groups (*p* < 0.04). During sympathetic activation, the cf-PWV was lower in women in H18–30 (*p* = 0.001), similar between sexes in H50–80 (*p* = 0.122), and higher in women in T2DM50–80 (*p* = 0.004). These data do not support the hypothesis that aortic stiffness increases in the morning compared to the evening within any of the considered groups in both rest and sympathetic activation conditions. There are differences between the sexes, which vary according to age and diabetes status. In particular, aortic stiffness is higher in older women than in men with diabetes during acute stress.

## Introduction

Circadian variations in physiological functions allow organisms to provide adequate physiological responses to recurring daily needs (1). Circadian clocks have been shown to be associated with cardiovascular functions (1, 2). Interestingly, the incidence of stroke, myocardial infarction, arrhythmia, and sudden cardiac death is higher in the morning compared to the evening (1, 2). A 40% higher risk of heart attack, a 29% increased risk of cardiac death, and a 49% increased risk of stroke have been suggested in the early morning between 6 a.m. and 12 a.m. (2). Endothelial function, an important index of cardiovascular risk, is blunted in the early morning compared to the evening (3, 4), whereas peripheral vascular resistance and blood pressure (BP) are increased (5). Augmented sympathetic activation in the morning may be a cardiovascular risk factor (4, 5). Interestingly, overt sex differences in the prevalence and severity of cardiovascular disease, as well as in sympathetic neurovascular modulation, have been documented (6–8). Cardiovascular disease has different symptomatology and response to treatment in one sex compared to the other (6). The incidence of acute cardiovascular events is higher in men compared to women of reproductive age; however, this trend starts reversing after menopause (9).

Among the possible contributors to the different incidence of acute cardiovascular events in the morning compared to the evening, as well as between sexes, greater aortic stiffness might be involved. Aortic stiffness is a compelling predictor of all-cause mortality (10). Aortic stiffness is an independent predictor of fatal stroke in patients with essential hypertension (11). Circadian variations of carotid-femoral pulse wave velocity (cf-PWV), the gold-standard measure to assess aortic stiffness, have been documented (4, 12, 13). Augmented aortic stiffness increases the afterload, work, and oxygen demand of the heart, as well as increases BP and pulse pressure (14). Augmented sympathetic outflow to the heart and blood vessels in the morning may also increase the risk of acute cardiovascular events by augmenting cardiac afterload and pulse pressure and reducing baroreflex sensitivity (5, 14).

The incidence of acute cardiovascular events normally increases with aging. However, the presence of type 2 diabetes (T2DM) increases such an occurrence (1, 15). T2DM leads to changes in central autonomic control and deleterious organ adaptations (15–17). High blood sugar and insulin in individuals with T2DM determine early-functional changes and remodeling of autonomic pathways controlling circulation, affecting cardiac and vascular cellular targets and feedback baroreceptor system sensitivity (15, 17, 18). Acute and chronic high blood insulin levels in individuals with T2DM augment sympathetic dominance, plasma catecholamines, and efferent sympathetic drive to the heart (17, 19). Imbalanced autonomic outflow toward the heart and vascular tissue has been associated with several pathological states, including cardiac autonomic neuropathy and deleterious cardiac remodeling in individuals with T2DM (19, 20). T2DM-induced remodeling has been found in vascular tissue and is associated with augmented arterial stiffness, acute endothelial dysfunction, vascular hypertrophy in small arteries, and impaired responsiveness to vascular smooth muscle stimulants (21, 22). T2DM-induced changes in the cardiovascular system have been suggested to blunt the normal circadian rhythms of heart rate (HR) and BP, leading to a high incidence of hypertension, myocardial infarction, hospitalization, and death (19).

This study aims to compare circadian and sex differences in aortic stiffness in young, healthy individuals, old healthy individuals, and old individuals with T2DM at rest and during sympathetic activation. As the incidence of acute cardiovascular events is higher in the morning compared to the evening, it is hypothesized that aortic stiffness, assessed *via* cf-PWV, is greater in the morning compared to the evening. Moreover, it is hypothesized that cf-PWV is lower in young women compared to young men and that such sex differences disappear in the older groups. The endothelial function is blunted at 6 a.m. compared to at 9 p.m., suggesting increased cardiovascular risk during that morning time (3). Therefore, the cf-PWV assessment in our study has been performed according to such a timing schedule.

## Materials and methods

The cf-PWV assessment was performed on 90 participants. The subjects were a random sample of the population of Northern Italy and were enrolled through recruitment flyers scattered around the cities. Subjects were divided into three groups as follows: 30 healthy individuals from 18 to 30 years old (H18-30), 30 healthy individuals from 50 to 80 years old (H50–80), and 30 individuals with T2DM from 50 to 80 years old (T2DM50–80). All groups were sex-balanced. All participants met common inclusion (>18 years old) and exclusion criteria (chronic hypertension, atrial fibrillation, cardiac valve disease, not in sinus rhythm, pacemaker-dependent, known significant carotid or femoral artery stenosis, an impalpable arterial pulse at the site of measurement, use of beta-blockers and ACE-inhibitors, pregnancy or presumed pregnancy). The specific inclusion criteria for healthy young and elderly subjects consisted of having fasting blood glucose lower than 100 mg/dL and being free of any cardiovascular, metabolic, neurological, or respiratory disease. The specific inclusion criteria for subjects with T2DM consisted of having been diagnosed with T2DM for at least 1 year. Specific exclusion criteria for subjects with T2DM consisted of severe autonomic neuropathy, pre-proliferative and proliferative retinopathy, and renal failure (23). The experiment was performed at the Cardiovascular Physiology Laboratory, School of Sports Science, University of Verona. We followed the recommendations regarding the management of the participants and laboratory presented in the study by Otto et al. (3). Complete silence was present in the laboratory throughout the tests. The laboratory temperature was set to 25° C. Participants were instructed not to consume caffeinated foods or drinks for 24 h and not to smoke for 8 h prior to testing. Participants were recommended to have at least 8 h of sleep. Participants visited our laboratory three times (preliminary visit; morning measures; and evening measures). Experiments were performed at 6 a.m. and 9 p.m., as previously done by Otto et al. (3). For each group, 50% of the subjects performed the second and third sessions on the same day. In contrast, the other subjects performed the second and third sessions in the evening and the following morning, respectively. The sample size was calculated according to the primary endpoints of our study, which were the circadian and sex differences in cf-PWV within each group. The information on changes in systolic BP, diastolic BP, and HR has a secondary role within our study and plays an exploratory role. The number of subjects was initially calculated through an a priori analysis of sample size (GPower 3.1.9.7; Universität Düsseldorf, Germany) using data on circadian and sex differences in cf-PWV at rest retrieved in previous investigations. The analysis was repeated after having collected data from 10 participants (five men and five women) within each group to evaluate any possible sample size adjustment to achieve a statistical power >80% at rest and during sympathetic activation. It was obtained that (H18–30: *n* = 10 and 12; H50–80: *n* = 16 and 20; T2DM50–80: *n* = 18 and 22; the number of subjects at rest and during sympathetic activation, respectively) were needed to assess circadian differences in cf-PWV, while (H18–30: *n* = 16 and 20; H50–80: *n* = 22 and 26; T2DM50–80: *n* = 24 and 28; the number of subjects at rest and during sympathetic activation, respectively, to be equally divided between men and women) were needed to assess sex differences. The sample size of 30 individuals per group we recruited is greater than that suggested by statistical analysis and close to or greater than that of similar previous investigations (4, 12, 13). The study was approved by the University of Verona Ethics Board (3293CESC) and conducted in accordance with the Declaration of Helsinki. Informed oral and written consent was obtained from all participants before starting any test.

### Experimental protocol

At the preliminary visit, our medical team assessed whether participants met the inclusion or exclusion criteria through physical examination, cardiovascular screening, and medical history review. Moreover, subjects completed two maximal handgrips (Saehan SH5001, Germany) contractions with their left hand to assess their maximum voluntary contraction. Each contraction lasted approximately 3 s and was separated by 4 m of rest. In the second and third sessions, participants laid supine on an ambulatory bed throughout the experiment. Participants were told to stay relaxed, breathe regularly, and not to speak throughout the experiment. Participants were suited with the electrocardiograph of the pulsed Doppler ultrasound machine (LOGIQ S7 pro, GE, Milwaukee, USA), as well as with a beat-by-beat finger BP and HR monitoring system (Finapres Medical System BV, The Netherlands) on the third medial phalanx of the right hand. After 10 m of supine and quiet rest, 3 BP measurements were taken using the Riva-Rocci method on the left arm and averaged to obtain systolic and diastolic BP values to calibrate the Finapres device. After further 10 m, the experiment started.

The protocol consisted of 5 m of rest followed by 5 m of acute sympathetic activation triggered by handgrip exercise at 30% of maximum voluntary contraction (24). The cf-PWV, systolic and diastolic BP and HR were measured during the last minute of each condition. The cf-PWV assessment was performed on the right side of the body, following the previously indicated user procedures guidelines (25). Details about the cf-PWV assessment *via* Doppler Ultrasound are reported in our previous paper (26). Briefly, scanning of the carotid artery at the supraclavicular level followed by another scan of the common femoral artery in the groin were performed. Measures were performed in B-mode with a pulsed Doppler Ultrasound with a Linear Array (6.6 MHZ) probe synchronized with ECG. The pulse transit time calculation was performed offline using the software installed on the ultrasound scanner. The software required manually placing the first cursor at the R peak of the ECG signal and a second cursor at the foot of the Doppler flow to return the time elapsed between the two points. The foot of the Doppler flow wave identifies the point where the steep rise of the waveform starts, as previously shown by Calabria et al. (27). The R-to-flow wave times at the carotid and femoral arteries were calculated on 15 consecutive cardiac cycles and then averaged to obtain the mean carotid and femoral pulse transit times, respectively. The carotid-femoral pulse transit time was then calculated as the absolute value of the difference between the mean carotid and femoral pulse transit times. The pulse transit distance was calculated as 0.8 times the length from the common carotid artery to the common femoral artery at the groin (25). Finally, the cf-PWV was calculated as pulse transit distance divided by carotid-femoral pulse transit time.

### Statistics

Within each group, circadian variations in cf-PWV, systolic BP, diastolic BP, and HR at rest were identified *via* a paired *t*-test by comparing the data collected in the morning vs. evening. The average value between the morning and evening values of the previous variables at rest was then calculated for each subject to assess sex differences. Within each group, sex differences in cf-PWV, systolic BP, diastolic BP, and HR at rest were identified *via* an unpaired *t*-test by comparing the data collected in the men vs. women. Rest and sympathetic activation were considered two independent conditions. Thus, statistical analyses were repeated with the data collected during sympathetic activation. The analysis of covariance required to assess the effects of systolic BP, diastolic BP, and HR on cf-PWV in the morning compared to the evening and in men compared to women was performed with MATLAB (MathWorks, USA). Significance was set at *p* < 0.05. GraphPad Prism 8 (GraphPad Software, San Diego, United States) was used for statistical analysis and graphs.

## Results

### Characteristics of the subjects

Table 1 shows the characteristics of the subjects. The mean age in H50–80 was similar to that in T2DM50–80. Body weights and BMIs were lower in H18–30 compared to H50–80 but similar in H50–80 compared to T2DM50–80. The mean duration of T2DM from diagnosis in the T2DM50–80 group was 6.2 ± 4.8 years.

**Table 1:** Characteristics of the subjects (*n* = 30), sex balanced within each group.

	**H18-30**		**H50-80**		**T2DM50-80**		***H18-30* vs. *H50-80***	***H50-80* vs. *T2DM50-80***
	**Men**	**Women**	**Men**	**Women**	**Men**	**Women**		
Age (years)	23.0 (4.0)	23.4 (3.1)	66.1 (7.1)	66.9 (7.4)	67.3 (7.5)	66.5 (8.1)	*p* <0.001	*p* = 0.964
Weight (Kg)	73.6 (7.1)	57.2 (8.1)*	76.5 (10.0)	70.3 (9.4)	86.1 (14.9)	79.4 (22.0)	*p* = 0.049	*p* = 0.101
Height (m)	1.80 (0.05	1.67 (0.07*	1.71 (0.05	1.61 (0.05*	1.77 (0.2	1.59 (0.07*	*p* = 0.32	*p* = 0.805
BMI (Kg/m^2^)	22.6 (1.6	20.5 (2.1*	26.2 (2.9	27.1 (4.2	27.6 (5.2	31.2 (7.7	*p* <0.001	*p* = 0.159
^#^Systolic BP (mmHg)	122.9 (7.4)	107.9 (9.3)*	136.9 (9.4)	128.6 (13.2)	144.4 (13.7)	140.2 (11.4)	*p* <0.001	*p* = 0.02
^#^Diastoli c BP (mmHg)	68.4 (5.5)	67.2 (8.3)	81.0 (4.6)	78.1 (6.9)	82.4 (8.9)	82.6 (8.9)	*p* <0.001	*p* = 0.34
^#^HR (bpm)	68.3 (7.6)	68.2 (11.2)	62.2 (8.5)	59.6 (8.2)	63.5 (7.2)	68.0 (6.0)	*p* <0.01	*p* = 0.13
^#^cf-PWV (m/s)	7.3 (1.0)	6.1 (0.5)*	7.7 (1.0)	8.8 (1.2)*	7.8 (0.9)	8.9 (1.4)*	*p* <0.001	*p* <0.001

*Compared to men; #Average value between morning and evening measure at rest. Data are reported as mean (SD).

### Circadian variations

**Figure 1:**
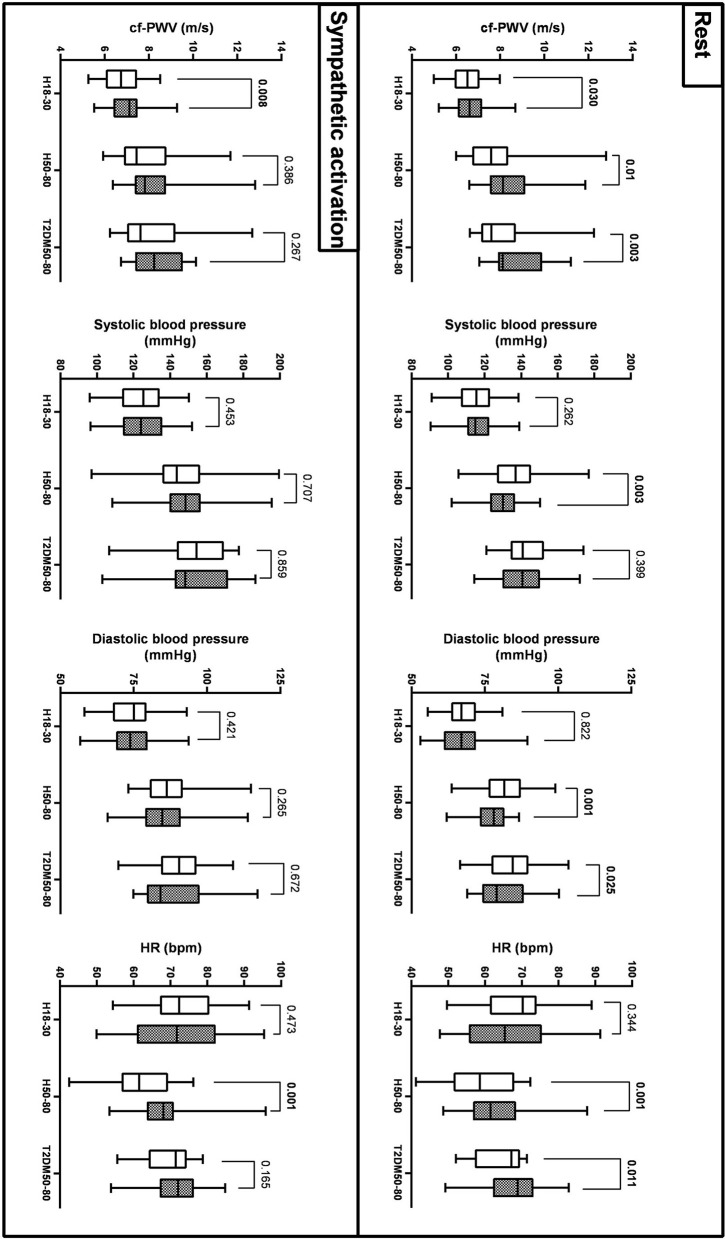
Circadian variations (morning: empty bars; evening: filled bars) of cf-PWV, systolic AP, diastolic AP, and HR across the 3 groups at rest and during sympathetic activation (mean, Q1/4, Q3/4, and minimum and maximum values).

As reported in Figure 1 and Table 2, cf-PWV values were lower in the morning compared to the evening in all groups at rest, while they were lower in H18–30 and similar in H50–80 and T2DM50–80 during sympathetic activation. Circadian differences in cf-PWV disappeared after adjusting for systolic BP, diastolic BP, and HR in all groups, both at rest and during sympathetic activation. Systolic BP values at rest were higher in the morning compared to the evening in H50–30 and similar in the other groups, while there were no circadian differences in all three groups during sympathetic activation. Diastolic BP was higher in the morning compared to the evening in H50–80 and T2DM50–80 but similar in H18–30 at rest, while no circadian differences were observed during sympathetic activation. HR was lower in the morning compared to the evening in H50–80 and T2DM50–80 but similar in H18–30 at rest, while it was similar in H18–30 and T2DM50–80 but lower in H50–80 during sympathetic activation.

**Table 2:** Circadian changes in cf-PWV at rest and during sympathetic activation within each group.

**Cf-PWV (m/s)**		**Morning**	**Evening**	***p*-value**	**Adjusted *p*-value**
Rest	H18-30	6.5 (0.8)	6.8 (1.0)	*p* = 0.03	*p* > 0.19
	H50-80	7.9 (1.6)	8.5 (1.3)	*p* = 0.01	*p* > 0.28
	T2DM50-80	8.0 (1.4)	8.6 (1.2)	*p* = 0.003	*p* > 0.18
Sympathetic	H18-30	6.8 (1.0)	7.2 (1.1)	*p* = 0.01	*p* > 0.19
activation	H50-80	8.2 (1.4)	8.7 (1.4)	*p* = 0.39	*p* > 0.43
	T2DM50-80	8.4 (1.5)	8.7 (1.1)	*p* = 0.27	*p* > 0.31

Data are reported as mean(SD). The p-value was adjusted for systolic BP, diastolic BP, and HR.

### Sex differences

**Figure 2:**
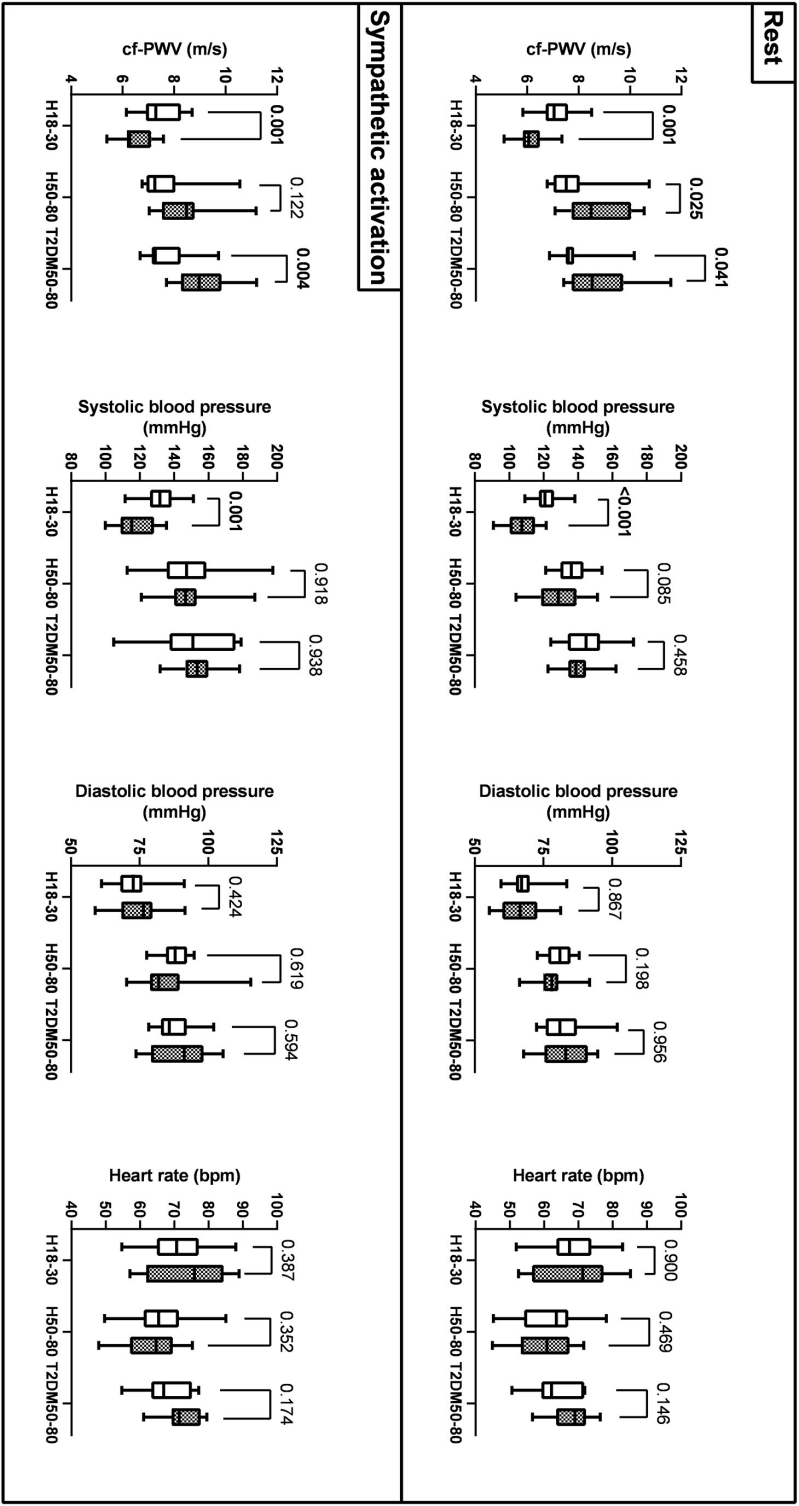
Sex differences (men: empty bars; women: filled bars) in cf-PWV, systolic AP, diastolic AP, and HR across the three groups at rest and during sympathetic activation (mean, Q1/4, Q3/4, and minimum and maximum values).

As reported in Figure 2 and Table 3, at rest, cf-PWV values were lower in young women compared to young men. However, this trend was reversed in the older groups. During sympathetic activation, the cf-PWV was still lower in young women but similar between sexes in H50–80 and higher in women compared to men in T2DM50–80. Sex differences in cf-PWV did not change after adjusting for systolic BP, diastolic BP, and HR in all groups, both at rest and during sympathetic activation. At rest and during sympathetic activation, systolic BP values were higher in the morning compared to the evening in H18–30 and similar in H50–80 and T2DM50–80. At both rest and sympathetic activation conditions, diastolic BP and HR were similar in the morning compared to the evening in all groups.

**Table 3:** Sex differences in cf-PWV at rest and during sympathetic activation within each group.

**Cf-PWV (m/s)**		**Men**	**Women**	***p*-value**	**Adjusted *p*-value**
Rest	H18-30	7.2 (0.7)	6.1 (0.5)	*p* = 0.001	*p* <0.001
	H50-80	7.7 (1.0)	8.8 (1.2)	*p* = 0.025	*p* <0.04
	T2DM50-80	7.8 (0.9)	8.9 (1.4)	*p* = 0.041	*p* <0.05
Sympathetic	H18-30	7.6 (1.0)	6.4 (0.6)	*p* = 0.001	*p* <0.03
activation	H50-80	8.0 (1.1)	9.0 (1.1)	*p* = 0.122	*p* > 0.08
	T2DM50-80	7.9 (1.4)	9.2 (1.2)	*p* = 0.004	*p* <0.01

Data are reported as mean(SD). The p-value was adjusted for systolic BP, diastolic BP, and HR.

## Discussion

We investigated circadian variations and sex differences in aortic stiffness assessed *via* cf-PWV in young healthy individuals, old healthy individuals, and old individuals with T2DM. Measures were performed in the early morning and the evening, at specific times when endothelial function, a compelling index of coronary artery disease and cardiovascular risk, is significantly different (3). Data were collected at rest and during sympathetic activation triggered by a standardized external stressor. Specifically, the static handgrip exercise we used to activate the sympathetic nervous system has been shown to reliably increase muscle sympathetic nerve activity (28) and peripheral vasoconstriction (24). It has been used in previous studies to evaluate the role of the sympathetic nervous system in regulating aortic stiffness (29, 30). Augmented aortic stiffness is an independent risk factor for cardiovascular events (10, 11). Chronically, augmented aortic stiffness can induce deleterious remodeling of the heart and vessels due to the greater cardiac afterload, leading to conditions such as heart failure and end-organ damage (10, 11, 31). Increased sympathetic outflow resulting from aging and disease can further increase aortic stiffness and contribute to deleterious cardiovascular effects (31). Interestingly, increases in sympathetic outflow have been associated with cardiac compensatory mechanisms in the presence of impaired cardiac hemodynamics due to cardiovascular disease (31). The focused study of the effects of the sympathetic nervous system on the heart has allowed us to identify relevant indices of myocardial dysfunction that may have prognostic implications in cardiovascular disease (31).

### Circadian variations

Contrary to our working hypothesis, at rest, the cf-PWV was lower in the morning compared to the evening within all the experimental groups. A previous study showed that aortic stiffness is generally lower at night (mean value from 6.30 p.m. to 6 a.m.) than during the day (mean value from 6.30 a.m. to 6 p.m.) in healthy middle-aged subjects of both sexes (13). When specific times of the day were chosen, however, another study showed that circadian variations in cf-PWV are similar in healthy young individuals and healthy elderly people at 9 a.m. compared to 5 p.m. (12). A study performing circadian comparisons at specific times when the endothelial function is blunted, as evinced by impaired brachial artery flow-mediated vasodilation in the morning, showed that cf-PWV is lower in old individuals with hypertension at 7 a.m. compared to 9 a.m. (4). To the best of our knowledge, no previous studies have performed such investigations in healthy young subjects, healthy older individuals, or older people with T2DM. Overall, the lower values of cf-PWV in the morning compared to the evening we found within all these groups at rest agree with most previous studies. Hence, aortic stiffness appears not to be increased in the morning at specific times while endothelial function has been suggested to be blunted. Moreover, lower resting cf-PWV in the morning is present in groups dissimilar in age and the presence of T2DM. At present, no study has assessed circadian variations in cf-PWV during sympathetic activation. The circadian variations in cf-PWV observed at rest did not change during sympathetic activation in H18–30, while they disappeared in H50–80 and T2DM50–80, in which cf-PWV values became similar in the morning compared to the evening. Thus, aortic stiffness appears not to be increased in the morning compared to the evening, even in the presence of sympathetic activation within any of the three groups examined. The cf-PWV is dependent on BP and HR (32). Thus, normalization of BP and HR was performed to identify changes in arterial stiffness independently of confounding factors. Interestingly, circadian variations in cf-PWV disappeared after adjusting for BP and HR in all groups, suggesting that circadian changes in aortic stiffness may largely be explained by variations in these variables between morning and evening. Furthermore, when present, circadian changes in cf-PWV were small (<0.6 m/s); therefore, it is of little concern from a clinical point of view. Similarly, the mean value of cf-PWV reached during sympathetic activation was only slightly increased compared to that at rest (<0.4 m/s). Circadian variations in BP and HR were absent in H18–30 but present in H50–80. BP was higher, and HR was lower in the morning compared to the evening in H50–80. Overall, the T2DM50–80 group showed similar circadian differences compared to those observed within the H50–80 group, except for a lack of circadian change in systolic BP.

### Sex differences

At rest, our cf-PWV data suggest that aortic stiffness is lower in women compared to men in young individuals and that this trend was reversed in older individuals without and with T2DM. Our data are consistent with previous literature suggesting that aortic stiffness is lower in young women than in young men after puberty and that women experience a more rapid increase in artery stiffening with aging (33). Among the causes, a key role for estrogen in the aging-associated increases in aortic stiffening in women has been suggested (33). The higher cf-PWV values in women with T2DM than in men with T2DM also agree with previous studies. Indeed, increases in cf-PWV have been suggested to occur mainly in women with T2DM rather than in men with T2DM (34). During sympathetic activation, the cf-PWV was still lower in women compared to men in young individuals. However, cf-PWV values became similar between the sexes in H50–80 and persisted higher in women compared to men in T2DM50–80. Overall, these findings suggest that aortic stiffness is higher at rest and similar under stress conditions in women compared to men in old healthy individuals and higher in women in both rest and stress conditions in old individuals with T2DM. Sex differences in cf-PWV persisted after adjusting for BP and HR, suggesting that a different aortic stiffness between sexes is independent of the diverse BP and HR values in men compared to women. Other factors could be responsible for different aortic stiffness between the sexes at different ages, including differences in the mechanical proprieties of the vessel or different sympathetic neurovascular transduction (7, 8). Systolic BP was higher in men than in women in H18–30 but similar in H50–80 and T2DM50–80. Although women show lower BP values compared to men at a young age, they display a steeper increase in BP than men, which starts in the third decade and continues through the life course, even if corrected for multiple cardiovascular disease risk factors (35). This may nullify sex differences in BP in adulthood that are otherwise present at a young age.

## Conclusion

Cf-PWV values were lower in the morning compared to the evening within all groups at rest, while they were lower in H18–30 and similar in H50–80 and T2DM50–80 during sympathetic activation. Hence, aortic stiffness appears not to be increased in the morning compared to the evening at specific times when the endothelial function has been suggested to be blunted, regardless of the presence of a stressful condition. At rest, cf-PWV values were lower in young women than in young men. However, this trend was reversed in the older groups. During sympathetic activation, the cf-PWV was still lower in young women but similar between the sexes in H50–80 and higher in men in T2DM50–80. Thus, older women have greater aortic stiffness than older men at rest, regardless of T2DM. While healthy older women show similar aortic stiffness values compared to their male counterparts during acute stress, older women with T2DM may have greater aortic stiffness than men with T2DM.

## Data availability statement

The raw data supporting the conclusions of this article will be made available by the authors, without undue reservation.

## Ethics statement

The studies involving human participants were reviewed and approved by Ethics Board of the University of Verona (3293CESC). The patients/participants provided their written informed consent to participate in this study.

## Author contributions

AG: study concept, design, subject recruitment, data acquisition, analysis, formal analysis, investigation, and original draft preparation. AG, AC, PM, AM, FS, and CT: data interpretation, review and critical revision of the manuscript. All authors read and approved the final version of this manuscript.

## Conflict of interest

The authors declare that the research was conducted in the absence of any commercial or financial relationships that could be construed as a potential conflict of interest.

## Publisher's note

All claims expressed in this article are solely those of the authors and do not necessarily represent those of their affiliated organizations, or those of the publisher, the editors and the reviewers. Any product that may be evaluated in this article, or claim that may be made by its manufacturer, is not guaranteed or endorsed by the publisher.
